# “If I could read your mind…”: parental mentalizing in mothers with borderline personality disorder

**DOI:** 10.1186/s40479-025-00290-7

**Published:** 2025-05-16

**Authors:** Jana Zitzmann, Anna Georg, Charlotte Rosenbach, Babette Renneberg

**Affiliations:** 1 Department of Education and Psychology, Institute for Clinical Psychology and Psychotherapy, Freie Universität Berlin, Berlin, Germany; 2 Centre for Psychosocial Medicine, Institute for Psychosocial Prevention, Heidelberg University Hospital, Heidelberg, Germany; 3 Department of Psychology, Institute for Clinical Psychology and Psychotherapy of Childhood and Adolescence, Universität Tübingen, Tübingen, Germany; 4 Department of Health, Health and Medical University Erfurt, Erfurt, Germany

**Keywords:** Borderline personality disorder, Anxiety disorder, Depressive disorder, Parenting, Mentalization, Reflective functioning, Mind-mindedness, FMSS

## Abstract

**Background:**

Individuals with a borderline personality disorder (BPD) show impairments in their ability to mentalize. Particularly in the parent-child relationship, mentalizing is an important foundation for sensitive parenting and the quality of interactive behavior. Previous studies of parental mentalizing in mothers with BPD are scarce and have focused primarily on one aspect of the multidimensional construct. In addition, there is currently no research comparing different mental disorders on different aspects of parental mentalizing, leaving disorder-specific differences unclear. Aim of this study is to examine disorder-specific differences in reflective functioning and mind-mindedness, two facets of parental mentalizing.

**Methods:**

We compared mothers with BPD (*n =* 156) with a clinical control group of mothers with depressive or anxiety disorders (*n =* 65) and with healthy mothers (*n =* 91) using non-parametric inference for multivariate data. Mothers completed the Parental Reflective Functioning Questionnaire (PRFQ) and participated in a five-minute speech sample (FMSS) in which they reflected on their child and their relationship with their child. Verbal transcripts of the FMSS were rated using an adapted manual for coding mind-mindedness with the FMSS that incorporates the assessment of additional characteristics of mind-related speech.

**Results:**

Mothers with BPD showed the highest impairments in parental mentalizing compared to both other groups, as evident in both operationalizations: They made more maladaptive attributions (PRFQ pre-mentalizing) than the other two groups and reported lower interest and curiosity and certainty in mental states than healthy mothers. In addition, mothers with BPD used more mental attributes with negative valence when asked to describe their child and the relationship compared to both other groups and more self-related mental attributes compared to healthy mothers. Additionally, Pearson correlational analyses revealed that only the use of mental attributes with negative valence was associated with all three subscales of the PRFQ in the anticipated directions. This supports the idea that the two operationalizations target different facets of parental mentalization.

**Conclusions:**

Our findings revealed impaired parental mentalization in several domains for mothers with BPD. Disorder-specific differences were observed in the amount of maladaptive attributions and in the negativity of mental state references. These aspects should be considered in diagnostic and therapeutic processes when working with mothers with BPD. As a limitation, it should be noted that the group comparisons did not control for sociodemographic variables, which may have contributed to some of the observed group differences.

## Background

Social interactions in our daily lives require us to understand and interpret the internal states of our interaction partner, such as thoughts, feelings, and desires that underlie behavior, as well as our own internal world. This ability is referred to as mentalization and is theorized to be a key socio-cognitive ability that influences the formation of our social relationships [1, 2].

Research on mentalization has increased in recent decades, with findings suggesting strong links to personality functioning [3]. Impairments in the ability to mentalize have been observed across a wide range of mental disorders (e.g., [4, 5]) suggesting its role as transdiagnostic vulnerability factor for mental health problems [6]. Research findings on mentalization point to a multidimensional concept with two poles on each dimension: Automatic versus controlled mentalizing, mentalizing regarding the self versus the interactional partner, mentalizing based on external versus internal aspects, cognitive versus affective mentalizing. Mental disorders are assumed to have characteristic mentalizing profiles, with individual patterns of imbalances regarding the dimensions. While balanced mentalization integrates an activation of both poles when needed, imbalances are characterized by reliance on one pole [6].

Clinical reports, together with behavioral and neurobiological research, suggest that mentalizing in people with borderline personality disorder (BPD) is fast, automatic and affect-driven, making it prone to error. In contrast, mentalizing about the self and the interaction partner appears to be impaired in individuals with depressive or anxiety disorders [6]. Mentalization based therapy focuses on improving mentalization and has shown efficacy in the treatment of chronic depression and BPD [7], further supporting relevance of mentalization as an underlying aspect in these disorders.

Mentalization is of high relevance in the parent-child relationship. Research has shown that parental mentalizing plays a crucial role in sensitive parenting and for the quality of interactive behavior (e.g., [8, 9]). Moreover, parental mentalizing is related to several aspects of child development, such as infant-parent attachment and child social-cognitive development (e.g., [10, 11]). There is evidence that parents with a mental disorder (e.g., BPD, depressive disorders) show impairments in their mentalizing capacity when interacting with or reflecting upon their children [6, 12, 13]. In a recent systematic review [13] on parental mentalizing in mothers with BPD and depression, both disorders as well as their symptom severity were associated with impairments in maternal mentalizing capacities. Similarly, Georg, Meyerhöfer [12] found in their meta-analysis that depression was associated with decreased levels of parental mentalizing. Particularly these results suggested a larger negative correlation in studies including parents with a diagnosis of depression, as opposed to studies that focused on symptom levels. Nevertheless, this evidence was limited as there were only four studies involving parents with a diagnosis of depression.

In the context of parental mentalizing, two operationalizations derived from different theoretical backgrounds [14] have been employed predominantly: Parental reflective functioning, and mind-mindedness [11, 12]. Consistent with the assumption that they encompass different facets of the multidimensional concept mentalization, previous research supports a differentiated perspective on different measures, observing significant differences across mentalization constructs [12, 13]. Disorder specific patterns of imbalances in mentalizing as suggested by Luyten, Campbell [6] could similarly apply to parental mentalizing. However, comparisons between several mental disorders regarding distinct profiles in parental mentalizing are rare [13]. To our knowledge, previous studies have only compared mothers with mental health problems to healthy mothers while primarily focusing on one aspect of parental mentalizing, limiting the investigation of potential disorder specific differences [12, 13]. Further investigation is required to determine whether and to what extent different facets of parental mentalizing are restricted as a function of different types of mental health difficulties. The present study addressed this question, with a focus on mothers with BPD, mothers with depressive or anxiety disorders, and healthy mothers.

### Parental reflective functioning and parental psychopathology

Parental reflective functioning describes caregivers’ ability to reflect on and acknowledge their child’s and their own inner world as a basis for behavior [15]. According to Luyten, Nijssens [16], three key aspects of parental reflective functioning are: (1) a non-mentalizing stance, often reflected in maladaptive attributions (pre-mentalizing); (2) certainty about the child’s mental states while acknowledging the opacity of mental states; (3) an interest and curiosity in the child’s mental states. The three facets are addressed in the Parental Reflective Functioning Questionnaire (PRFQ; [15]), an instrument frequently used in previous studies.

Individuals with BPD have been rarely studied [13] with the PRFQ and research yielded slightly inconsistent results. In a recent study on parents with personality disorder (50% of them diagnosed with a BPD), Hestbaek, Kretzschmar [17] observed significantly higher pre-mentalizing and lower certainty about mental states compared to healthy parents. No group difference was found for interest and curiosity between healthy parents and parents with personality disorder. Steele, Townsend [18] reported parents with elevated BPD features to show increased pre-mentalizing and certainty about mental states compared to those with low BPD features. In another study of mothers with postpartum depression, no significant association was found between the three aspects of parental reflective functioning and a comorbid BPD [19].

In a recent meta-analysis on parents with diagnosed depression and depressive symptoms, Georg, Meyerhöfer [12] found a small-sized association between higher depression scores and reduced parental reflective functioning, with the effect size being largest for pre-mentalizing compared to interest and curiosity or certainty in mental states. A positive correlation between symptom levels of depression and pre-mentalizing was consistently observed across studies (e.g., [19–21]). The findings regarding certainty about mental states have been less consistent. Two studies observed a negative association between depressive symptoms and certainty about mental states [20, 21], whereas another study found no such association [19]. With regard to interest and curiosity, no association with depression symptoms was observed across studies [19–21].

In the studies that addressed anxiety, pre-mentalizing was observed to be consistently associated with higher symptom levels [19, 21]. Only one of the studies [21] observed an association between certainty about mental states and anxiety symptoms. Again, for interest and curiosity no significant associations were observed [19, 21].

### Mind-mindedness and parental psychopathology

According to Meins, Fernyhough [22], mind-mindedness refers to the “Caregivers’ tendency to treat infants as intentional agents” ([22], p. 1194) by acknowledging the infant as an individual with a mind and considering the child’s inner world. The representational approach conceptualizes mind-mindedness as a spontaneous tendency to utilize mental attributes when speaking freely about the child, suggesting that elevated mind-related speech reflects enhanced mentalizing capacity. Additionally, the observational approach focuses on the appropriateness of spontaneously used mental state references during interaction with the child. Non-attuned mind-related comments reflect an incorrect interpretation of a child’s behavior due to an impaired awareness of the child’s perspective [23].

In the study of mind-mindedness among individuals with BPD, there are findings that clearly demonstrate a relation with some mind-mindedness parameters. It is important to note, however, that the number of studies in this area is limited [13], and no recent publications have been published in the last few years. Marcoux, Bernier [24] observed that mothers with BPD did not differ from healthy mothers in terms of the quantity of mind-related speech or the use of appropriate comments. However, they demonstrated a significantly higher frequency of non-attuned mind-related comments when interacting with their children. Using the representational approach, Schacht, Hammond [25] found mothers with BPD to use significantly less mind-related speech compared to healthy mothers. However, after adjusting for depressive symptoms, group differences were not significant anymore.

For individuals with depressive disorders or symptoms findings are less consistent [12, 13]. In their meta-analysis, Georg, Meyerhöfer [12] found partly reduced levels of mind-mindedness only in parents with clinically relevant levels of depression, but not in at-risk samples. In community mothers, depressive symptoms were not associated with the amount of mind-related speech when participating in an interview about the child [26, 27]. While one study found significantly fewer appropriate mind-related comments among inpatient mothers with depressive disorder compared to healthy mothers [28], other studies found no significant association between appropriate or non-attuned mind-related comments and depressive symptoms [19, 29].

For anxiety disorders, evidence is scarce. Fishburn, Meins [26] found significant negative associations between anxiety symptoms and mind-related speech in an interview measure among community mothers. Similarly, trait anxiety was associated with more non-attuned mind-related comments during free-play [29, 30].

A key limitation of the representational approach to assessing mind-mindedness is that it focuses solely on the quantity of mind-related speech, without capturing qualitative aspects. As mentioned above, research using the observational approach has highlighted qualitative impairments in mind-mindedness among parents with psychopathology, particularly in BPD [24]. However, the observational approach is only applicable within a narrow age range (up to 12 months), whereas the representational approach allows for assessment across a broader developmental period [31].

Given the specific characteristics of BPD – including heightened emotional reactivity, difficulties in mentalizing under stress [32], a tendency toward hyper- or hypomentalizing [16], and a negativity bias [33, 34] – these factors may also shape how parents represent and describe both their child and themselves in the context of parenting. Additionally, mentalization theory emphasizes the importance of uncertainty regarding mental state awareness [1], aspects not captured by traditional mind-mindedness coding. Hence, a more nuanced approach to coding mind-mindedness in the context of parental psychopathology is warranted. To address this gap, we adapted and extended the original manual by incorporating additional indices that capture further characteristics of mind-related speech. These new indices aim to assess aspects particularly relevant for understanding mind-mindedness in parents with BPD and, more broadly, in the context of parental psychopathology.

### Association between parental reflective functioning and representational mind-mindedness

Although research points to a differentiated view, there is at the same time a conceptual overlap in parental reflective functioning and representational mind-mindedness. Both constructs assess parental mentalizing regarding the child at a representational level (see [11, 35]). Items in the PRFQ and the request in the interview to describe the child (representational mind-mindedness) require parents to actively reflect their explicit convictions about their child’s thoughts and feelings. The crucial difference is, the mind-mindedness interview measure targets spontaneous mentalization and is observer-based while the PRFQ relies on self-ratings and is limited on the item content.

In their study, Smith-Nielsen, Stuart [36] employed a modified mind-mindedness coding scheme to assess the frequency of mental state language used by community mothers during free play. They found that mothers who used more mental state language scored higher on interest and curiosity in the PRFQ. Similarly, a greater utilization of mental descriptors in the mind-mindedness interview was found to be positively associated with parental reflective functioning, as assessed via the Parent Development Interview (PDI; [37]). A higher level of parental reflective functioning is indicated by a higher level of complexity and coherence in the description of the child [38]. To our knowledge, there is no research on the association between PRFQ and representational mind-mindedness and none in clinical samples. A combined investigation of both measures in a single study may yield new insights into the various facets of parental mentalizing and their potential association.

### Aim of the current study

Evidence suggests that parental mentalizing may be impaired in parents with mental health conditions, though findings vary by psychopathology and by assessment method [6, 12, 13]. Research in clinical samples is limited, but mothers with BPD show consistently reduced parental mentalizing, including elevated pre-mentalizing (PRFQ; [39]) and reduced mind-mindedness [25]. For parents with clinically relevant depression or anxiety symptoms, higher levels of pre-mentalizing (PRFQ) are commonly observed, while evidence on mind-mindedness is scarce and inconsistent (e.g., [12, 26]). Furthermore, there is currently no research comparing different mental disorders regarding different aspects of maternal mentalizing, leaving disorder-specific differences unclear.

This study aims to analyze such differences among mothers with BPD, mothers with anxiety or depression, and a healthy control group. In accordance with the understanding of parental mentalizing as a multidimensional construct with potential individual impairments depending on psychopathology [6], we will examine different facets of parental mentalizing using two distinct operationalization approaches: Parental reflective functioning, as assessed by the PRFQ, and representational mind-mindedness, as evaluated through the analysis of interview transcripts. Building on theoretical considerations and prior research [40, 41], we expanded the original mind-mindedness manual for evaluating mind-mindedness to enable a more comprehensive examination of particular aspects of mind-related speech (Georg A, Zitzmann J: Manual Mind-Mindedness Kodierung von Reflexionsaufgaben mit dem FMSS, unpublished).

Our primary research question is whether there are differences in the two conceptualizations of parental mentalization between mothers with BPD, mothers with anxiety or depressive disorder, and healthy mothers. Based on theoretical assumptions and previous research findings we hypothesize (1) that mothers with BPD and mothers with anxiety or depressive disorder will show lower levels of parental reflective functioning compared to healthy mothers; (2) that mothers with BPD will show lower levels of adapted mind-mindedness compared to mothers with anxiety or depressive disorder and healthy mothers. In order to gain preliminary insight into the relationship between the two operationalizations, PRFQ and adapted mind-mindedness, we will additionally explore the association between them.

## Methods

We conducted a cross-sectional study. Data were drawn from the study “Parenting Skills for Mothers with BPD” [42], that is part of the ProChild consortium (“Preventing maltreatment and promoting mental health in children of mothers with borderline personality disorder”), funded by the Federal Ministry of Education and Research (001KRI805A). The study is designed as a randomized, controlled, multicenter trial and aims to evaluate a group intervention and to identify the specific characteristics of mothers with BPD (M-BPD) compared to mothers with anxiety disorder or depressive disorder (M-A/D) and healthy mothers (M-HC). Detailed information on study design, recruitment, and eligibility can be found in Rosenbach, Heinrichs [42]. For this study, we used baseline data of the three study groups before the intervention started. The study groups were selected according to the aims of the primary study [42]. Consequently, the clinical control group comprised two highly prevalent mental disorders with the aim to reach a broader impact. This secondary data analysis has been pre-registered (10.17605/OSF.IO/H2MF7).

### Procedure and participants

After a telephone screening for inclusion and exclusion criteria (see [42]), all mothers visited the research laboratory to participate in the Structured Clinical Interview for DSM- 5 [43, 44], and in a five-minute speech sample (FMSS; [45]), in which they reflected on their child and their relationship with their child. Sociodemographic information was collected through a self-administered interview.

Three hundred twelve mothers (*n* = 156 M-BPD, *n* = 65 M-A/D, *n* = 91 M-HC) with a mean age of 33.7 years (*SD* = 5.9; range 17 to 52 years) and at least one child aged between six months and seven years participated in this part of the research project. In the clinical groups, over half of the mothers reported that they were currently undergoing outpatient treatment (M-BPD: *n* = 97, 62.2%; M-A/D: *n* = 38, 58.5%). Furthermore, almost half of the participants were currently taking psychotropic medication on a regular basis (M-BPD: *n* = 76, 48.7%; M-A/D: *n* = 32, 49.2%). For the M-HC group, current psychotherapeutic or psychiatric treatment was an exclusion criterion.

If the mothers had more than one child in the required age range, they were asked to select one child as an index child, preferably the child they felt was the most challenging to parent. The assessments were then made in relation to this index child. The index children were on average 38.4 months old (*SD* = 22.4; range 6 to 90 months) and had on average 0.6 siblings (*SD* = 0.8; range 0 to 5). Half of the index children were female (*n* = 165; 52.9%).

After the laboratory visit, mothers completed web-based questionnaires from home. The electronic data collection tool REDCap [46] was used for data collection and management.

### Measures

#### Parental reflective functioning

Mothers completed the PRFQ [15], a self-report measure on parental reflective functioning of parents with a child up to five years of age that consists of 18 items and three subscales. The subscales assess three key aspects of parental reflective functioning: (1) Pre-mentalizing (PRFQ_PM_; e.g., “My child cries around strangers to embarrass me.”) represents the amount of maladaptive mentalizing; (2) Certainty about mental states (PRFQ_CMS_; e.g., “I can completely read my child’s mind.”) in an extremely high amount can be understood as hypermentalizing or as a lack of a non-knowing stance, which, in contrast, is assumed to reflect a higher mentalizing capacity. A complete lack of certainty, as indicated by extremely low values, is seen as an expression of hypomentalizing; (3) Interest and curiosity (PRFQ_IC_; e.g., “I try to see situations through the eyes of my child.”) is suggested to be an important aspect of adaptive mentalizing while low levels are seen as absence of interest [15, 16]. The items are answered on a 7-point Likert scale, ranging from 1 (“strongly disagree”) to 7 (“strongly agree”). Means for the three subscales were calculated, with higher values reflecting higher scores on the respective scale.

Although previous research has supported the validity of the PRFQ [15], the internal consistencies of the subscales have been found to vary considerably and, in some cases, to be questionable (e.g., [47]). In our sample, Cronbach’s alphas coefficients for PRFQ_PM_, PRFQ_CMS_, and PRFQ_IC_ were 0.70, 0.77, and 0.65, respectively, which is slightly higher than in previous research (e.g., [48, 49]).

As noted by Luyten, Mayes [15], children over the age of five years display further progress in the development of more advanced language skills and a greater comprehension of the social world. Consequently, parental mentalizing increasingly relies on internal features of the child, possibly encompassing different mentalization processes than those captured by the PRFQ. However, the reliability and validity of the PRFQ have recently been supported in a study of mothers with children aged three to 11 years [50]. Since only *n* = 28 children in our sample were older than the proposed age range and no alternative questionnaire covering the full age range of our participants was available, we decided to use the PRFQ.

In an exploratory analysis, consistent with the recommendation of Anis, Perez [51], we squared the deviations from the sample mean for the PRFQ_CMS_ and PRFQ_IC_ subscales (score = [y - mean]^2^). Higher scores indicate a higher deviation from the sample mean and thus lower levels of parental reflective functioning (range 0–36). This method is consistent with the theoretical assumption that moderate scores may reflect good levels of parental reflective functioning, whereas extremely low or high scores may indicate reduced capacity [16]. However, most of the studies use the scoring system initially proposed by Luyten, Mayes [15]. Descriptive statistics of PRFQ for the total sample and the subgroups can be found in Table 1.

**Table 1:** Descriptive statistics of PRFQ subscales and adapted mind-mindedness indices by group

	Total (*N =* 312) M (SD) [Min, Max]	M-BPD (*n =* 156) M (SD) [Min, Max]	M-A/D (*n =* 65) M (SD) [Min, Max]	M-HC (*n =* 91) M (SD) [Min, Max]
**Parental reflective functioning**				
PRFQ_PM_	1.97 (0.87) [1.00, 6.00]	2.33 (0.93) [1.00, 6.00]	1.82 (0.70) [1.00, 4.67]	1.47 (0.50) [1.00, 3.00]
PRFQ_CMS_	3.47 (1.07) [1.00, 6.50]	3.29 (1.06) [1.17, 6.50]	3.54 (1.00) [1.00, 5.67]	3.73 (1.09) [1.50, 6.17]
PRFQ_IC_^a^	5.69 (0.84) [1.33, 7.00]	5.53 (0.88) [1.33, 7.00]	5.77 (0.79) [3.67, 7.00]	5.90 (0.75) [3.17, 7.00]
PRFQ_CMS−T_	1.14 (1.41) [0.00, 9.19]	1.15 (1.41) [0.00, 9.19]	0.99 (1.34) [0.00, 6.09]	1.23 (1.45) [0.00, 7.28]
PRFQ_IC−T_^a^	0.70 (1.38) [0.00, 19.00]	0.79 (1.77) [0.00, 19.00]	0.62 (0.81) [0.00, 4.09]	0.60 (0.84) [0.00, 6.36]
**Adapted mind-mindedness (relative)**^b^				
MM	2.79 (1.11) [0.38, 8.79]	2.85 (1.09) [0.38, 5.71]	2.84 (1.36) [0.90, 8.79]	2.67 (0.95) [0.52, 5.99]
MM_self_	0.72 (0.81) [0, 4.59]	0.83 (0.85) [0, 4.59]	0.75 (0.913) [0, 4.53]	0.50 (0.58) [0, 3.25]
MM_child_	2.08 (0.99) [0, 5.86]	2.01 (1.02) [0, 5.36]	2.09 (1.10) [0.19, 5.86]	2.18 (0.85) [0.52, 4.95]
MM_nk_	9.30 (11.11) [0, 100]	9.03 (12.30) [0, 100]	7.73 (8.70) [0, 37.50]	10.9 (10.50) [0, 46.20]
MM_pos_	14.50 (12.00) [0, 62.50]	14.80 (12.50) [0, 60.00]	13.40 (12.00) [0, 62.50]	15.00 (11.00) [0, 47.40]
MM_neg_	5.22 (9.17) [0, 50.00]	7.70 (10.40) [0, 50.00]	4.37 (9.62) [0, 42.90]	1.57 (3.83) [0, 28.60]
MM_neutral_	80.10 (14.50) [26.90, 100]	77.40 (16.00) [26.90, 100]	82.30 (13.30) [37.50, 100]	83.40 (11.30) [52.60, 100]
**Adapted mind-mindedness (frequency)**				
MM_nr_	16.80 (6.43) [2.00, 36.00]	16.30 (6.14) [2.00, 36.00]	16.70 (6.74) [4.00, 34.00]	17.80 (6.66) [3.00, 35.00]
MM_self_nr_	4.31 (4.56) [0, 22.00]	4.81 (4.78) [0, 19.00]	4.31 (4.79) [0, 22.00]	3.45 (3.89) [0, 19.00]
MM_child_nr_	12.50 (5.63) [0, 27.00]	11.40 (5.23) [0, 27.00]	12.40 (5.88) [1.00, 27.00]	14.40 (5.68) [3.00, 27.00]
MM_nk_nr_	1.53 (1.95) [0, 18.00]	1.49 (2.14) [0, 18.00]	1.22 (1.26) [0, 4.00]	1.84 (1.99) [0, 12.00]
MM_pos_nr_	2.39 (2.06) [0, 14.00]	2.36 (2.09) [0, 11.00]	2.03 (1.45) [0, 6.00]	2.70 (2.32) [0, 14.00]
MM_neg_nr_	0.92 (1.81) [0, 13.00]	1.38 (2.13) [0, 13.00]	0.72 (1.87) [0, 13.00]	0.26 (0.51) [0, 2.00]
MM_neutral_nr_	13.50 (5.56) [2.00, 29.00]	12.60 (5.05) [2.00, 24.00]	13.90 (5.93) [2.00, 26.00]	14.80 (5.89) [3.00, 29.00]

*M-BPD* mothers with borderline personality disorder, *M-A/D* mothers with anxiety or depressive disorder, *M-HC* healthy mothers, *PRFQ* parental reflective functioning questionnaire, *PRFQ*_*PM*_ pre-mentalizing, *PRFQ*_*CMS*_ certainty about mental states, *PRFQ*_*IC*_ interest and curiosity, *PRFQ*_*CMS−T*_ certainty about mental states, transformed score: (y = mean)^2^, *PRFQ*_*IC−T*_ interest and curiosity, transformed score: (y = mean)^2^, *MM*_*(nr)*_ mind-mindedness, *MM*_*self(_nr)*_ related to self, *MM*_*child(_nr)*_ related to child, *MM*_*nk(_nr)*_ not-knowing stance, *MM*_*pos(_nr)*_ positivity, *MM*_*neg(_nr)*_ negativity, *MM*_*neutral(_nr)*_ neutrality

^a^*n =* 155 M-BPD

^b^These values have been multiplied by a factor of 10

#### Adapted mind-mindedness

Mind-mindedness was coded using a manual for coding mind-mindedness with the FMSS (Georg A, Zitzmann J: Manual Mind-Mindedness Kodierung von Reflexionsaufgaben mit dem FMSS, unpublished) that was adapted specifically for this study. This manual is based on the concept and manual for coding mind-mindedness, version 2.2 [31] and the manual for coding reflective tasks, version 2.6 (Georg A, Bruno L, Taubner S, Hausschild S: Manual Mind-mindedness Kodierung von Reflexionsaufgaben, Version 2.6, unpublished). The original approach [31] captures maternal mind-mindedness as a mother’s tendency to use mental attributes when speaking freely about her child [52]. Prior research has indicated the construct validity of the original mind-mindedness approach (comparable to the MM_child_ index in the here applied manual) and found associations consistent with mentalization theory like for example sensitivity [53] and child’s theory of mind performance [22].

Recent studies have successfully assessed mind-mindedness and reflective functioning based on the FMSS (e.g., [41, 54]). The adapted manual (Georg A, Zitzmann J: Manual Mind-Mindedness Kodierung von Reflexionsaufgaben mit dem FMSS, unpublished) employed in this study represents an extension of the original version, designed to be applicable to transcripts of the FMSS and to incorporate the assessment of additional characteristics of mind-related speech. With the goal of stimulating mentalization about the relationship, the instruction was adjusted according to the FMSS by including a reflection on the mother-child relationship (instruction: “I’d like to hear your thoughts and feeling about [CHILD], in your own words. I’d like you to speak for five minutes, telling me what kind of a person [CHILD] is and how the two of you get along together.”). As proposed by Fonagy and Luyten [55], the process of mentalization is facilitated by slight elevations in arousal levels. By describing the child and the mother’s perception of the relationship, it is assumed that emotional responses are elicited.

The manual for coding mind-mindedness with the FMSS (Georg A, Zitzmann J: Manual Mind-Mindedness Kodierung von Reflexionsaufgaben mit dem FMSS, unpublished) encompasses seven indices:

(1) MM is the frequency of mind-related attributes (e.g., “She *hates* it when…”) relative to the total number of words to control for verbosity (range 0–1). The index indicates the extent to which a mother is mind-minded when reflecting on her child and their relationship, with higher scores indicating more mind-mindedness.

Because mentalization processes encompass the appraisal of both one’s own mind and the mind of others [1], and building on the manual for coding reflective tasks (Georg A, Bruno L, Taubner S, Hausschild S: Manual Mind-mindedness Kodierung von Reflexionsaufgaben, Version 2.6, unpublished), we included separate scores in addition to a total score (MM) that includes both. (2) MM_self_ and (3) MM_child_ are the frequencies of mental attributes related to the self (e.g., “Sometimes I am *afraid* of losing her…”) and child (e.g., “He is *curious*…”), respectively, relative to the total number of words (range 0–1). While higher scores on the MM_self_ index indicate a greater extent to which a mother is mind-minded when reflecting on herself in relation to her child, MM_child_ represents reflection about the child. MM_child_ conceptually corresponds to the only existing mind-mindedness index from the original manual, however, it is based on slightly different material due to the use of the FMSS.

According to mentalization theory [1], a central aspect of the mentalizing ability is the awareness that we can only infer mental states and internal processes without knowing them with certainty. Therefore, and building on the manual for coding reflective tasks (Georg A, Bruno L, Taubner S, Hausschild S: Manual Mind-mindedness Kodierung von Reflexionsaufgaben, Version 2.6, unpublished), we included (4) MM_nk_ as the relative frequency of mental attributes mentioned in relation to words signaling an attitude of not knowing (e.g., “I *assume* he wants me to be more present…”) relative to the total number of mental attributes (range 0–1). The closer the value is to 1, the more mental attributes are reflected in the context of a not-knowing stance.

Given the repeatedly observed negativity bias in individuals with BPD by showing a tendency to make malicious attributions to neutral stimuli (e.g., [33, 34]), which is also prevalent in the context of parenting (e.g., [56, 57]), we wanted to take a closer look at whether mothers represent themselves and their child with positive or negative valence. As previously proposed by Demers, Bernier [40] and also incorporated into the adapted manual (Georg A, Zitzmann J: Manual Mind-Mindedness Kodierung von Reflexionsaufgaben mit dem FMSS, unpublished) used in this study, the emotional valence of mental attributes was rated as either positive (e.g., “He is *patient*…”), negative (e.g., “He is *stubborn*…”), or neutral (e.g.,” She *likes* to…”). To determine this rating, we considered how the mother experiences the mental attribute in relation to herself or her child. Specifically, the attribute is first assessed based on its explicit wording, and if this is inconclusive, the context in which the attribute is mentioned is then taken into account. If neither positive nor negative valence is indicated and uncertainty persists, the attribute is categorized as neutral. Maternal negativity ([5] MM_neg_), positivity ([6] MM_pos_), and neutrality ([7] MM_neutral_) mind-minded indices are calculated as proportional scores of mental attributes with negative, positive, or neutral emotional valence relative to the total number of mental attributes (range 0–1).

During the pilot phase, four raters (JZ, AG, and two undergraduate psychology students) tested and refined the adapted manual for coding mind-mindedness with the FMSS (Georg A, Zitzmann J: Manual Mind-Mindedness Kodierung von Reflexionsaufgaben mit dem FMSS, unpublished) by independently rating pilot data until satisfactory interrater reliability was achieved, defined as intraclass correlation coefficients (ICCs) of 0.80 or higher, indicating good interrater reliability according to the guidelines proposed by Koo and Li [58]. Disparities were discussed until consensus was reached. While the adapted manual already provided detailed descriptions of the above introduced mind-mindedness indices, step-by-step instructions, illustrative examples to guide the coding process, and guidelines for calculating indices, additional clarifications and guidelines were incorporated during piloting to address ambiguous or complex cases and enhance coding consistency.

In the subsequent coding phase, eight independent raters (undergraduate psychology students) were trained by JZ and AG. Raters completed a training dataset to achieve good interrater reliability (ICCs ≥ 0.80) before proceeding with the study data. FMSS audio recordings were transcribed verbatim by different undergraduate psychology students. To ensure interrater agreement during coding process, a randomly selected subset of the study data (*n* = 75; 25.34%) was double-coded by one rater. Any ambiguities and disparities were resolved through regular consultations and discussions with JZ and AG until consensus was reached. Raters coded blind to group status, the hypotheses of the study and other participant data.

The obtained ICCs indicate moderate (MM_neutral_: ICC = 0.69) to excellent (MM_child_: ICC = 0.92) reliability [58]. The interrater agreement for MM_child_ was higher than that observed in previous research (e.g., [25, 26]). However, for emotional valence (MM_neutral_), it was slightly lower than that previously documented (e.g., [27, 40]). The interrater agreement for MM_self_ and MM_nk_ was comparable to that reported in a previous study utilizing the coding of reflective tasks [41], although this study was not conducted in a parenting context. The seven indices introduced above were included in the analyses. Table 1 presents the descriptive statistics of the mind-mindedness indices for the total sample and the subgroups.

In addition to the indices computed relative to maternal verbosity, as recommended in coding manuals and implemented in the majority of previous studies [31, 59], it may also be relevant to consider the total number of mental attributes. Some studies employ these frequency scores (e.g., [60]) and posit that parents must be able to mentalize in order to express mental attributes. It is proposed that children hear these comments regardless of what their parents otherwise say, thereby making an influence on the child’s development conceivable [59–61]. Therefore, exploratory analyses were conducted using the frequency scores of the above introduced mind-mindedness coding categories (e.g., MM_nr_ is the total number of mental attributes used by a mother). The descriptive statistics of frequency scores are presented in Table 1.

### Analyses

Analyses were performed in RStudio (version 2024.04.2 + 764; [62]). Prior to hypothesis testing, descriptive statistics were examined for all variables. One single missing value was identified for the PRFQ, and therefore the corresponding subscale means (PRFQ_IC_ and PRFQ_IC−T_) were not calculated for this mother. The sociodemographic characteristics of the three study groups were compared using chi-squared tests for categorical variables. Mosaic plots and residual analyses were used to identify specific differences between groups in the categories of each variable. Kruskal-Wallis tests and post-hoc Dunn tests were used to compare numerical variables because they were not normally distributed.

To answer our research question, we planned to conduct two different Multivariate Analyses of Variance (MANOVA), because mind-mindedness and parental reflective functioning are considered theoretically related but distinct constructs. The prerequisites for a MANOVA were checked beforehand. A review of the box plots revealed the presence of outliers for the PRFQ subscales and the mind-mindedness indices. The inclusion of outliers in the analysis was deemed necessary to ensure that the full range of variability and potentially meaningful patterns within the data set were captured. Removal of these cases could have introduced bias or missed important findings. Due to the lack of multivariate and univariate normal distribution indicated by Q-Q plots and the heterogeneity of the covariance matrices indicated by Box’s M-tests, we decided not to proceed with the MANOVAs. We chose to use non-parametric inference for multivariate data (R package *npmv*), which does not require multivariate normality of the data [63].

Two separate non-parametric tests were conducted, with group as the independent variable and the PRFQ subscales (Hypothesis 1) or the adapted mind-mindedness indices (Hypothesis 2) as the dependent variables. Wilks’ Lambda was chosen as the test statistic. For statistically significant results in the multivariate analyses, post-hoc non-parametric Kruskal-Wallis tests were performed for each dependent variable, and post-hoc Dunn tests were performed for group comparisons (M-BPD, M-A/D, and M-HC). Results were corrected for multiple comparisons using the Bonferroni correction. Depending on the test statistic, Epsilon squared (ε^2^) and Cohen’s d were calculated as effect sizes and interpreted according to Cohen [64].

Pearson correlation analyses were used to determine the relationship between the adapted mind-mindedness indices and the PRFQ subscales. Finally, intercorrelations among study variables and sociodemographic variables were calculated using Pearson’s correlation coefficient for metric variables (age mother, age index-child) and point-biserial correlation coefficient for categorical variables (sex index-child, education mother).

The *p* <.05 criterion was employed in all analyses.

The sample sizes were selected to ensure sufficient power for the randomized, controlled, multicenter trial. Accordingly, their suitability for the secondary analyses conducted here is not guaranteed. To avoid the loss of any data, we elected to include all of them.

## Results

### Participant characteristics

The children of M-BPD were significantly older than the children of M-HC (*Z*_BPD−HC_ = 2.63, *p* =.026). The mothers’ reported level of education ranged from no school degree (*n* = 8; 2.6%) to having a university degree (*n* = 119; 38.1%), with a substantial proportion reporting higher education levels. M-BPD reported a significantly higher frequency of lower education levels (residual = 3.96) and a significantly lower frequency of higher education levels (residual = − 2.94). In contrast, M-HC were significantly more likely to have obtained a higher level of education (residual = 2.92) and were less likely to have obtained a lower level of education (residual = − 3.93). Finally, M-BPD used significantly less words in the FMSS than M-HC (*Z*_BPD−HC_ = 3.91, *p* <.001) and M-A/D (*Z*_BPD−AD_ = 3.14, *p* =.005). The sociodemographic data for the total sample and the subgroups, together with the results of the group comparisons, are presented in Table 2.

**Table 2:** Relevant sociodemographic data by group

	Total (*N =* 312)	M-BPD (*n =* 156)	M-A/D (*n =* 65)	M-HC (*n =* 91)	Test statistic
**Age mother (years)**					*H*(2) = 4.58, *p* =.101
* M (SD)*	33.7 (5.94)	32.9 (6.24)	34.1 (5.55)	34.5 (5.56)	
[Min, Max]	[17.0, 52.0]	[17.0, 48.0]	[22.0, 44.0]	[24.0, 52.0]	
**Age index-child (months)**					*H*(2) = 6.97, *p* =.031
* M (SD)*	38.4 (22.4)	41.3 (23.5)	38.8 (21.6)	33.0 (20.3)	
[Min, Max]	[6.00, 90.0]	[6.00, 90.0]	[6.00, 82.0]	[6.00, 77.0]	
**Sex index-child**					*χ*^2^(2) = 0.70, *p* =.703
Female, *n* (%)	165 (52.9%)	79 (50.6%)	35 (53.8%)	51 (56.0%)	
**Education mother**					*χ*^2^(2) = 51.75, *p* <.001
Lower, *n* (%)	111 (35.6%)	85 (54.5%)	16 (24.6%)	10 (11.0%)	
Higher, *n* (%)	201 (64.4%)	71 (45.5%)	49 (75.4%)	81 (89.0%)	
**Number of words in FMSS**					*H*(2) = 17.10, *p* <.001
* M (SD)*	621 (153)	595 (158)	607 (136)	676 (144)	
[Min, Max]	[188, 1020]	[188, 924]	[273, 1010]	[258, 1020]	

Lower education mother = no qualification, lower secondary education, secondary school degree; Higher education mother = high school degree, university degree

*M-BPD* mothers with borderline personality disorder, *M-A/D* mothers with anxiety or depressive disorder, *M-HC* healthy mothers

### Group differences in parental reflective functioning

To test our initial hypothesis that M-BPD and M-A/D will demonstrate lower levels of parental reflective functioning in comparison to M-HC, we employed a non-parametric inference approach to analyze the comparison of multivariate data samples. A statistically significant effect of group was observed on the combined dependent variables (PRFQ_PM_, PRFQ_CMS_, and PRFQ_IC_), *F*(6, 612) = 14.78, *p* <.001. Non-parametric relative effects are used to quantify the tendencies observed in the data, expressed in terms of probabilities [63]. The results indicate that M-BPD tend to report higher values in PRFQ_PM_ compared to the other groups (see Table 3). For example, the probability that a randomly chosen mother in this group reports higher values in PRFQ_PM_ than a randomly chosen mother from the full sample is 0.63.

**Table 3:** Probabilities of higher PRFQ subscale values by group (relative effects)

	PRFQ_PM_	PRFQ_IC_	PRFQ_CMS_
M-BPD	0.63	0.45	0.45
M-A/D	0.46	0.53	0.53
M-HC	0.31	0.57	0.57

*M-BPD* mothers with borderline personality disorder, *M-A/D* mothers with anxiety or depressive disorder, *M-HC* healthy mothers, *PRFQ* parental reflective functioning questionnaire, *PRFQ*_*PM*_ pre-mentalizing, *PRFQ*_*IC*_ interest and curiosity, *PRFQ*_*CMS*_ certainty about mental states

In a next step, we performed Kruskal-Wallis tests for each PRFQ subscale. They revealed statistically significant differences between the three groups in PRFQ_PM_ (*H*(2) = 71.50, *p* <.001, *ε*^2^ = 0.23), PRFQ_IC_ (*H*(2) = 11.30, *p* =.004, *ε*^2^ = 0.03), and PRFQ_CMS_ (*H*(2) = 10.50, *p* =.005, *ε*^2^ = 0.03) with large (PRFQ_PM_) to medium (PRFQ_IC_ and PRFQ_CMS_) effect sizes.

**Fig. 1:**
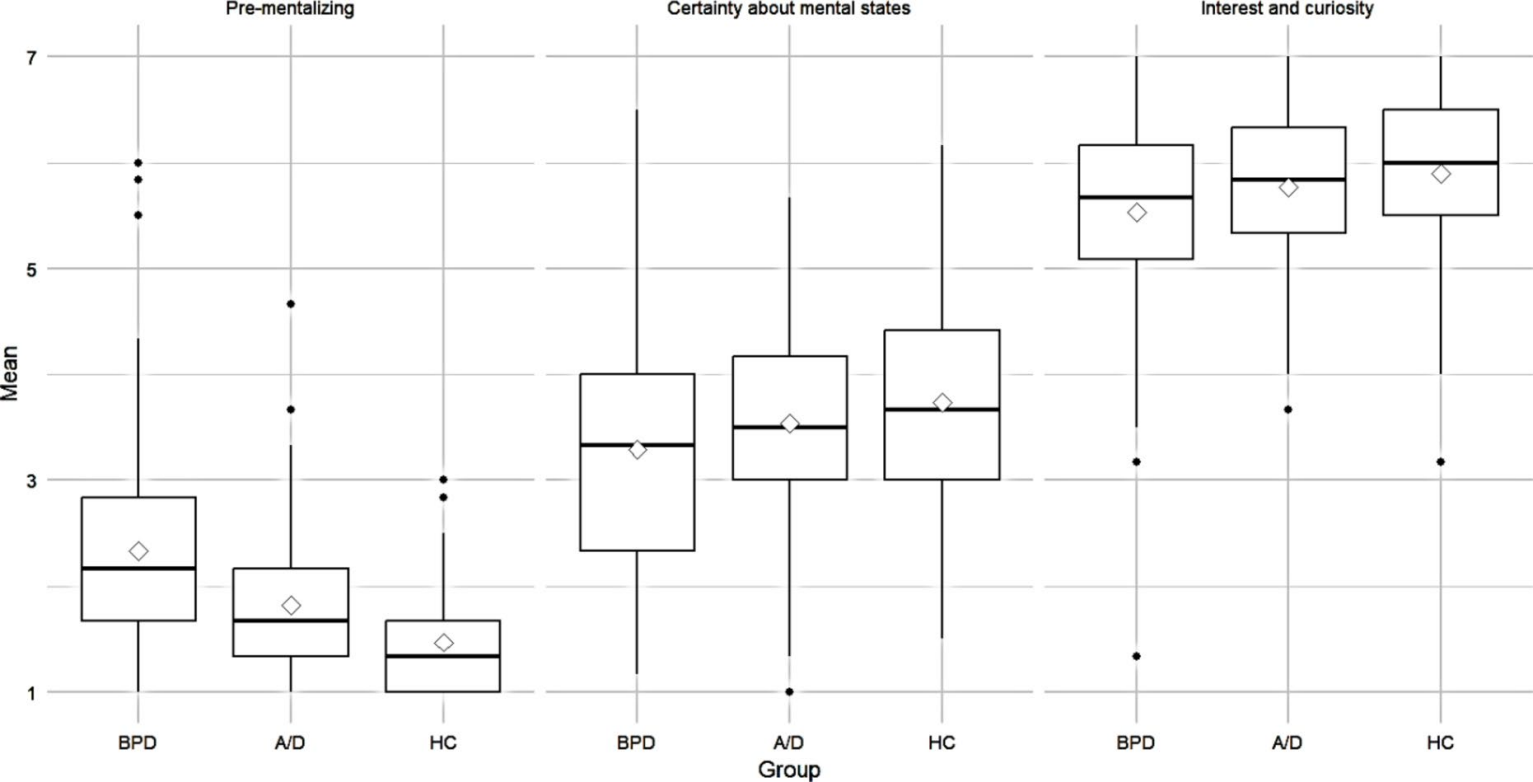
Boxplots for the PRFQ subscales by group

Post-hoc Dunn tests with Bonferroni correction revealed significant differences between all groups for PRFQ_PM_ (BPD vs. HC: *Z* = − 8.35, *p* <.001; BPD vs. A/D: *Z* = − 4.02, *p* ​< 0.001; A/D vs. HC: *Z* = − 3.12, *p* =.005) with large (*d*_BPD−HC_ = 1.08) to medium (*d*_BPD−A/D_ = 0.59, *d*_A/D−HC_ = 0.59) effect sizes. For PRFQ_IC_ (*Z* = 3.25, *p* =.003) and PRFQ_CMS_ (*Z* = 3.12, *p* =.005), post-hoc Dunn tests with Bonferroni correction indicate significant differences between M-BPD and M-HC with small effect sizes (*d*_Ic_ = 0.43, *d*_CMS_ = 0.42). Boxplots are shown in Fig. 1.

In the exploratory analysis, the squared deviations from the sample mean for the PRFQ_CMS_ and PRFQ_IC_ subscales were employed in accordance with the recommendations set forth by Anis, Perez [51]. A descriptive analysis revealed that M-BPD scored higher on both transformed scales (PRFQ_CMS−T_: max = 9.19, PRFQ_IC−T_: max = 19.00) than other groups (PRFQ_CMS−T_: max between 6.09 and 7.28, PRFQ_IC−T_: max between 4.09 and 6.36) and compared to the data reported by Anis, Perez [51] using the same transformation of the scores (PRFQ_CMS−T_: max = 6.84, PRFQ_IC−T_: max = 3.95). However, the Kruskal-Wallis tests indicated that there were no statistically significant differences between the three groups in PRFQ_CMS−T_ (*H*(2) = 1.15, *p* =.563) and PRFQ_IC−T_ (*H*(2) = 0.06, *p* =.970).

### Group differences in adapted mind-mindedness

To test the second hypothesis, that M-BPD will exhibit lower levels of adapted mind-mindedness than M-A/D and M-HC, a second non-parametric inference was calculated for the comparison of multivariate data samples. A statistically significant effect of group was observed on the combined dependent variables (MM, MM_self_, MM_child_, MM_nk_, MM_pos_, MM_neg_, MM_neutral_), *F*(14, 606) = 3.60, *p* <.001. The non-parametric relative effects indicate that M-BPD tend to have higher scores in MM_neg_ compared to the other groups (see Table 4). For example, the probability that a randomly chosen mother in this group has higher scores in MM_neg_ than a randomly chosen mother from the full sample is 0.58.

**Table 4:** Probabilities of higher mind-mindedness indices by group (relative effects)

	MM	MM_self_	MM_child_	MM_nk_	MM_pos_	MM_neg_	MM_neutral_
M-BPD	0.51	0.54	0.48	0.48	0.50	0.58	0.45
M-A/D	0.50	0.50	0.50	0.47	0.47	0.45	0.54
M-HC	0.48	0.43	0.54	0.55	0.52	0.39	0.56

*M-BPD* mothers with borderline personality disorder, *M-A/D* mothers with anxiety or depressive disorder, *M-HC* healthy mothers, *MM* mind-mindesness, *MM*_*self*_ related to self, *MM*_*child*_ related to child, *MM*_*nk*_ not-knowing stance, *MM*_*pos*_ positivity, *MM*_*neg*_ negativity, *MM*_*neutral*_ neutrality

The subsequent step involved the implementation of Kruskal-Wallis tests for each mind-mindedness index. The results demonstrate a statistically significant difference between the three groups in MM_self_ (*H*(2) = 7.74, *p* =.021) with a medium effect size (*ε*^2^ = 0.02), in MM_neg_ (*H*(2) = 35.40, *p* <.001) with a large effect size (*ε*^2^ = 0.11), and in MM_neutral_ (*H*(2) = 9.93, *p* =.007) with a medium effect size (*ε*^2^ = 0.03).

**Fig. 2:**
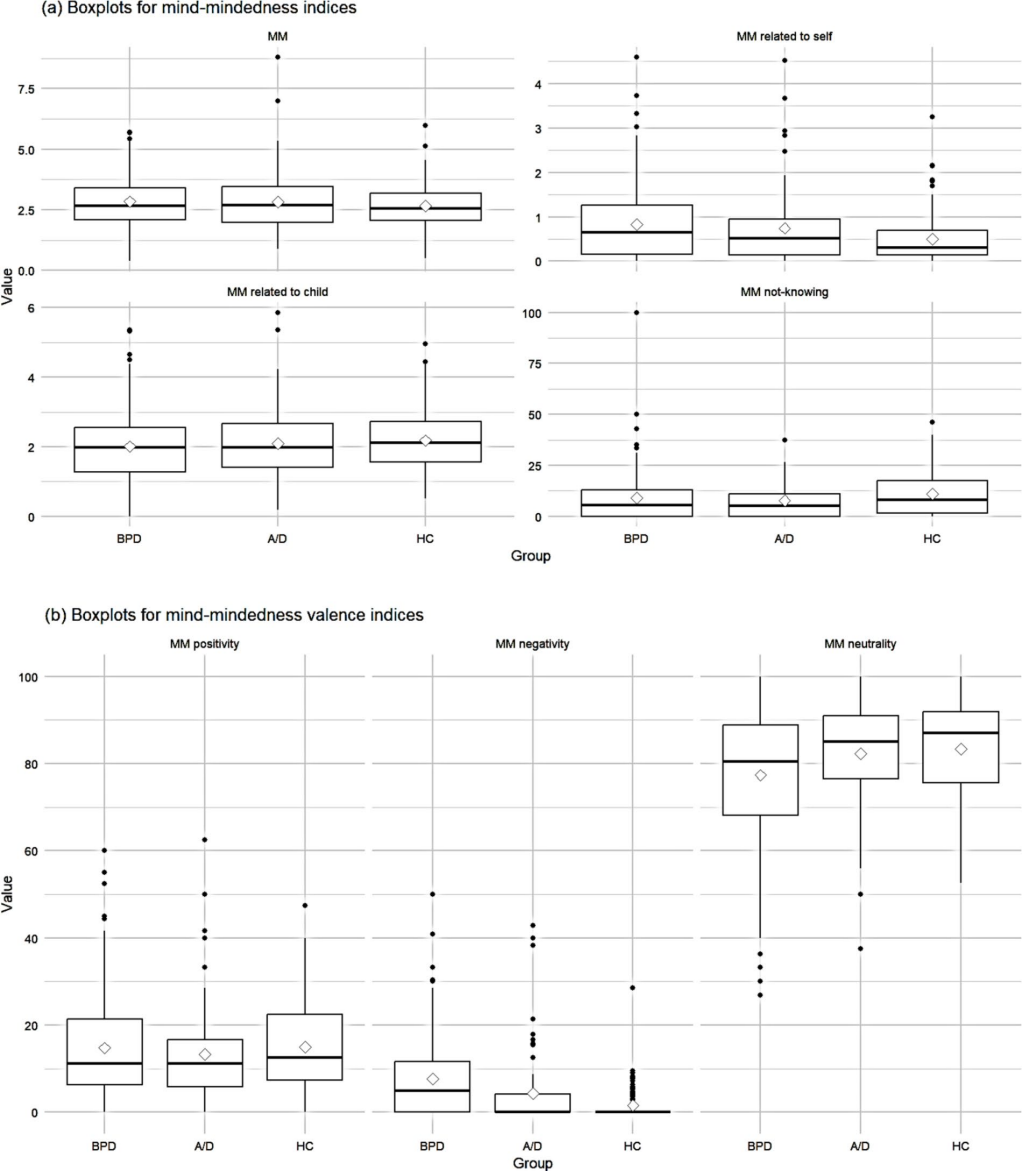
Boxplots for (**a**) the mind-mindedness indices and (**b**) the mind-mindedness valence indices by group

Post-hoc Dunn tests with Bonferroni correction revealed significant differences between M-BPD and M-HC for MM_self_ (*Z* = − 2.78, *p* =.016) with a small effect size (*d*_BPD−HC_ = 0.43). For MM_neg_, post-hoc Dunn tests with Bonferroni correction indicate significant differences between M-BPD and M-HC (*Z* = − 5.63, *p* <.001), as well as between M-BPD and M-A/D (*Z* = − 3.67, *p* <.001) with small (*d*_BPD−A/D_ = 0.33) to medium (*d*_BPD−HC_ = 0.72) effect sizes. For MM_neutral_, post-hoc Dunn tests with Bonferroni correction indicate significant differences between M-BPD and M-HC (*Z* = 2.84, *p* =.014) with small (*d*_BPD−HC_ = 0.42) effect size. Boxplots are shown in Fig. 2.

#### Negative valence in self and child references

In exploratory analyses, we conducted a more detailed examination of the tendency to utilize attributes with negative valence, either in reference to the child (MM_neg_child_ relative to MM_child_), or in regard to the mother herself (MM_neg_self_ relative to MM_self_).

A Kruskal-Wallis test indicates significant group differences for negativity in child references (*H*(2) = 6.63, *p* =.036, *ε*^2^ = 0.01). Post-hoc Dunn tests with Bonferroni correction indicate a marginally significant difference between M-BPD and M-HC (*Z* = − 2.39, *p* =.051, *d*_BPD−HC_ = 0.30) in the direction of greater negativity in child references among M-BPD (*M* = 0.03, *SD* = 0.06) compared to M-HC (*M* = 0.01, *SD* = 0.04).

With regard to negativity in self references, a significant group difference was observed (*H*(2) = 27.10, *p* <.001, *ε*^2^ = 0.08). Post-hoc Dunn tests with Bonferroni correction revealed significant differences between M-BPD and M-HC (*Z* = − 4.95, *p* <.001, *d*_BPD−HC_ = 0.58), as well as between M-BPD and M-A/D (*Z* = − 3.16, *p* =.005, *d*_BPD−A/D_ = 0.34) in the direction of greater negativity in self references among M-BPD (*M* = 0.18, *SD* = 0.26) compared to both other groups (*M*_A/D_ = 0.09, *SD*_A/D_ = 0.21; *M*_HC_ = 0.05, *SD*_HC_ = 0.15).

#### Mind-mindedness frequency scores

Using the total number of attributes instead of the relative values for the adapted mind-mindedness indices, we conducted further exploratory analyses. A statistically significant effect of group was observed on the combined dependent variables using a non-parametric inference for the comparison of multivariate data samples (MM_nr_, MM_self_nr_, MM_child_nr_, MM_nk_nr_, MM_pos_nr_, MM_neg_nr_, MM_neutral_nr_), *F*(14, 606) = 4.32, *p* <.001.

Results of Kruskal-Wallis tests for each absolute mind-mindedness index demonstrate a statistically significant difference between the three groups in MM_child_nr_ (*H*(2) = 13.90, *p* <.001) with a medium effect size (*ε*^2^ = 0.04), in MM_neg_nr_ (*H*(2) = 34.30, *p* <.001) with a large effect size (*ε*^2^ = 0.10), and in MM_neutral_nr_ (*H*(2) = 8.18, *p* =.716) with a medium effect size (*ε*^2^ = 0.02).

Post-hoc Dunn tests with Bonferroni correction revealed significant differences between M-BPD and M-HC for MM_child_nr_ (*Z* = 3.73, *p* <.001) with a medium effect size (*d*_BPD−HC_ = 0.55) in the direction of more child references among M-HC compared to M-BPD (see Table 1). For MM_neg_nr_, post-hoc Dunn tests with Bonferroni correction indicate significant differences between M-BPD and M-HC (*Z* = − 5.51, *p* <.001), as well as between M-BPD and M-A/D (*Z* = − 3.68, *p* <.001) with small (*d*_BPD−A/D_ = 0.32) to medium (*d*_BPD−HC_ = 0.65) effect sizes in the direction of higher negativity among M-BPD compared to both other groups. For MM_neutral_nr_, post-hoc Dunn tests with Bonferroni correction indicate significant differences between M-BPD and M-HC (*Z* = 2.71, *p* =.020) with small (*d*_BPD−HC_ = 0.42) effect size in the direction of more neutrality among M-HC compared to M-BPD.

### Association between parental reflective functioning, mind-mindedness, and sociodemographic variables

To examine the association between the PRFQ subscales and the adapted mind-mindedness indices, correlational analyses were employed. As illustrated in Table 5, there was a small positive correlation between the utilization of mental attributes with negative valence (MM_neg_) and self-reported pre-mentalizing modes (PRFQ_PM_). Moreover, there were small negative correlations between MM_neg_ and certainty about their child’s mental states (PRFQ_CMS_), and interest and curiosity in their child’s mental states (PRFQ_IC_). Finally, there was a small positive correlation between the utilization of mental attributes with positive valence (MM_pos_) and PRFQ_IC_. No significant association was observed between the other adapted mind-mindedness indices and the PRFQ subscales.

**Table 5:** Intercorrelations among PRFQ subscales, adapted mind-mindedness indices and sociodemographic variables in the overall sample

Variable	1	2	3	4	5	6	7	8	9	10	11	12
1. PRFQ_PM_	–											
2. PRFQ_CMS_	− 0.17^**^	–										
3. PRFQ_IC_	− 0.35^***^	0.18^**^	–									
4. PRFQ_CMS−T_	0.03	0.02	− 0.08	–								
5. PRFQ_IC−T_	0.29^***^	0.09	− 0.49^***^	0.24^***^	–							
6. MM	0.07	− 0.05	− 0.01	0.03	− 0.05	–						
7. MM_self_	0.06	− 0.10	0.04	0.08	− 0.08	0.50^***^	–					
8. MM_child_	0.02	0.03	− 0.04	− 0.03	0.01	0.71^***^	− 0.25^***^	–				
9. MM_nk_	− 0.04	− 0.01	0.03	− 0.02	− 0.02	− 0.10	− 0.07	− 0.06	–			
10. MM_pos_	− 0.11	0.06	0.12^*^	− 0.02	− 0.05	− 0.01	0.04	− 0.03	0.06	–		
11. MM_neg_	0.24^***^	− 0.22^***^	− 0.12^*^	0.03	− 0.01	0.10	0.38^***^	− 0.21^***^	0.03	− 0.08	–	
12. MM_neutral_	− 0.07	0.09	− 0.01	0.01	0.04	− 0.07	− 0.27^***^	0.15^**^	− 0.06	− 0.77^***^	− 0.57^***^	–
13. Age mother	− 0.02	− 0.06	− 0.15^**^	0.08	0.06	− 0.05	− 0.08	0.01	0.00	− 0.06	0.04	0.03
14. Age index-child	0.23^***^	− 0.08	− 0.15^**^	0.04	0.11	0.08	− 0.07	0.13^*^	− 0.05	− 0.11	0.03	0.07
15. Sex index-child	0.03	0.01	− 0.01	0.05	0.00	− 0.03	0.02	− 0.04	0.04	0.02	0.03	− 0.04
16. Education	− 0.23^***^	0.10	0.31^***^	0.07	− 0.23^***^	0.03	0.04	0.01	0.12^*^	0.04	− 0.05	0.00

Sex index-child is coded 0 for males, 1 for females

Education is coded 0 for lower maternal education, 1 for higher maternal education

*PRFQ*_*PM*_ pre-mentalizing, *PRFQ*_*CMS*_ certainty about mental states, *PRFQ*_*IC*_ interest and curiosity, *PRFQ*_*CMS−T*_ certainty about mental states, transformed score: (y = mean)^2^, *PRFQ*_*IC−T*_ interest and curiosity, transformed score: (y = mean)^2^, *MM* Mind-Mindesness, *MM*_*self*_ related to self, *MM*_*child*_ related to child, *MM*_*nk*_ not-knowing stance, *MM*_*pos*_ positivity, *MM*_*neg*_ negativity, *MM*_*neutral*_ neutrality

^*^p <.05

^**^p <.01

^***^p <.001

As anticipated, no substantial correlations were identified between the two operationalizations of parental mentalization, with the exception of emotional valence in mind-mindedness. This finding provides support for the analysis plan that involved examining both measurements separately in order to address the research question.

Additionally, we conducted correlational analyses to examine associations between both operationalizations of parental mentalization and sociodemographic variables. For the PRFQ subscales, self-reported pre-mentalizing modes (PRFQ_PM_) showed a small positive correlation with child age and a small negative correlation with maternal education. Furthermore, small negative correlations were found between self-reported interest and curiosity in the child’s mental states (PRFQ_IC_) and both maternal age and child age, while a moderate positive correlation emerged between PRFQ_IC_ and maternal education (see Table 5).

Regarding the adapted mind-mindedness indices, a small positive correlation was observed between the use of mental attributes related to the child (MM_child_) and child age, as well as between the use of mental attributes reflecting a not-knowing stance (MM_nk_) and maternal education (see Table 5).

## Discussion

This study aimed to examine parental mentalizing among M-BPD, comparing them with both clinical and healthy control groups across two different parental mentalization conceptualizations and using distinct operational approaches, namely parental reflective functioning and adapted mind-mindedness. Our goal was to determine whether disorder-specific differences in parental mentalization exist, as prior studies have predominantly focused on the comparison with healthy parents. Our findings revealed impaired parental mentalization across various domains for M-BPD, while M-A/D displayed only elevated pre-mentalizing (an aspect of reflective functioning). Indications for disorder-specific differences were observed in pre-mentalizing and negativity of mental state references. However, the observed group differences in parental mentalization may partly reflect sociodemographic differences between the groups. Specifically, the lower education levels and older child age among M-BPD may contribute to the observed effects, suggesting that these differences could partially reflect sociodemographic rather than exclusively mental health-related influences.

With the exception of negative emotional valence, the two operationalization approaches of parental mentalization were not significantly correlated. This finding lends support to the view that they represent distinct dimensions of the broader construct of mentalization [16].

### Group differences in parental reflective functioning

The PRFQ results partially confirmed our hypothesis that M-BPD and M-A/D would exhibit reduced levels of self-reported parental reflective functioning compared to M-HC. Mothers in the clinical groups exhibited a diminished capacity to engage in effective reasoning about the child’s intentions and a reduced ability to gain insight into the child’s internal world (as reflected by elevated pre-mentalizing modes in the PRFQ), indicating greater difficulties and distortions in their mentalizing capacity compared to M-HC. However, since M-BPD were characterized by lower educational levels and older child age compared to M-HC, and both sociodemographic factors were significantly associated with PRFQ_PM_, the observed differences may partially reflect sociodemographic rather than exclusively mental health-related influences. Although the PRFQ has been shown to be a reliable and valid measure for mothers of children up to the age of 11 [50], some aspects of parental mentalization may still be influenced by child age. It is possible that parental mentalization encompasses different processes for older children than those captured by the PRFQ, due to the child’s progress in the development of language skills and social understanding [15]. Similarly, maternal education may influence maternal mentalizing ability, as previous studies have also found associations with the PRFQ (particularly pre-mentalizing; [15]). According to Fonagy, Luyten [65], less favorable social learning conditions among individuals with lower education levels may contribute to the development of less accurate or more malevolent attributions when engaging in mentalizing.

Nevertheless, the observed group differences align with findings from previous research on parents with personality disorder [39], BPD features [18], and parents with depression or anxiety symptoms [12, 19–21]. It is noteworthy that M-BPD exhibited even higher pre-mentalizing modes than M-A/D, indicating that pre-mentalizing may not only be a characteristic of mental disorders in general, but may also be more pronounced among individuals with BPD.

Contrary to our initial hypothesis, only M-BPD, but not M-A/D, reported lower levels of interest and curiosity and less certainty about their child’s mental states, than M-HC, possibly reflecting aspects of hypomentalization. This suggests even greater differences between M-BPD and M-HC. However, since educational level and child age were associated with PRFQ_IC_ (but not with PRFQ_CMS_), these factors may additionally have contributed to the observed group differences.

Nevertheless, these findings may indicate that the diminished capacity for mentalization observed in individuals with BPD represents a broader limitation in personality functioning [3]. Conversely, it is possible that individuals with depressive or anxiety disorders may experience more nuanced or transient impairments in their ability to mentalize, contingent with the phases of the illness and the underlying processing mechanisms. With regard to processing mechanisms, it is evident that in BPD, compared to other disorders, there are specific deficits in various aspects of social cognition that may be associated with mentalization impairments [66], such as an attentional bias to negative verbal stimuli [67], rejection sensitivity [68, 69], as well as the communication and interpretation of social information [69]. Future research should examine which disease-immanent or secondary mechanisms are actually present in different psychopathologies and are associated with parental mentalization impairments.

No differences between groups were observed with respect to transformed scores based on deviations from the sample mean (PRFQ_CMS−T_, PRFQ_IC−T_). This might be due to the sample composition (e.g., 50% M-BPD), which influenced sample means and limited variability. Consequently, the observed means for both scores are in close proximity to those observed for M-BPD. Given that higher transformed scores reflect larger deviations from the sample mean (whether positive or negative), and M-BPD exhibited higher scores on both transformed scales on a descriptive level, this could suggest a tendency toward greater variability and reduced reflective functioning in M-BPD [51]. While speculative, this potential pattern of increased deviations may suggest that M-BPD are more prone to hypermentalizing (i.e., excessive certainty or intrusive interest; [16]) or hypomentalizing (i.e., excessive uncertainty or complete lack of interest; [16]). This interpretation corroborates prior research indicating the presence of both types of mentalizing difficulties in individuals with BPD (e.g., [70, 71]). However, since group differences were not statistically significant, this observation remains purely descriptive and inferences based on transformed scores should be drawn cautiously, as the sample composition likely influences score variability, limiting the generalizability of these results. Further research with more balanced samples or alternative scoring methodologies would offer invaluable insights into these patterns.

### Group differences in adapted mind-mindedness

To further investigate specific aspects of mind-related speech, we developed extensions to the original manual for the assessment of representational mind-mindedness [31]. This initial study applying the adapted manual for coding mind-mindedness with the FMSS (Georg A, Zitzmann J: Manual Mind-Mindedness Kodierung von Reflexionsaufgaben mit dem FMSS, unpublished) yielded promising results, as indicated by moderate to excellent interrater agreement and preliminary evidence of differences between the study groups in some facets of mind-mindedness in the expected direction. In this regard, our data partially support our hypothesis that M-BPD would display lower levels of adapted mind-mindedness than M-A/D and M-HC.

While the current findings provide valuable insights into the reliability of the additional mind-mindedness indices, they should be interpreted with caution due to the preliminary nature of this application. It is important to acknowledge that the indices require further validation through additional studies to establish their validity and generalizability, particularly by investigating their associations with related constructs and their predictive power in various contexts. As this study marks the initial use of the extended manual in the context of parenting, comparability with prior research findings is restricted to a certain degree. The MM_child_ index (the proportion of mental attributes referring to the child) exhibits the greatest conceptual alignment with the representational mind-mindedness index from earlier studies.

#### Self-related mental state speech

We observed that M-BPD demonstrated a markedly elevated proclivity to employ mental attributes pertaining to themselves in comparison to M-HC. This suggests that M-BPD may find it more straightforward to reflect on their own internal states than on those of their children when asked to reflect on their children and their relationship. From a clinical perspective, this observation is not unsurprising, as individuals with psychopathology tend to be more preoccupied with their own internal experiences. Furthermore, individuals with BPD exhibit a higher level of self-focus in studies on autobiographical memory [72]. Additionally, they tend to diffuse self-referential and other-referential states [73], which may result in a greater number of verbalized self-references during the interview. It would be beneficial for future studies to investigate the impact of this heightened self-focus in parental mentalization on parenting behavior.

#### Negativity

A greater use of mental attributes with negative valence was observed among M-BPD compared to both other groups, reflecting a more negative representation of the child and their common togetherness. We also observed reduced neutrality among M-BPD, likely at the expense of increased negativity. The results indicated that M-A/D did not exhibit a greater negativity than M-HC, which may point to a disorder-specific aspect of parental mentalization in M-BPD. This finding aligns with the well-documented negativity bias observed in individuals with BPD (e.g., [33, 34, 56, 57]).

Upon closer examination, it was observed that all groups tended to use similar levels of negative child-references, with only marginally more negative child-references in M-BPD compared to M-HC. However, M-BPD used more negative self-references, potentially reflecting a negative self-representation. Moreover, they might experience a greater degree of conflictual and more negative emotions in their parental role. These assumptions are consistent with the findings of ambulatory assessment studies on negative emotions and interpersonal stressors among individuals with BPD (e.g., [74]). Specifically in the context of parenthood, M-BPD report lower parenting efficacy [56, 75, 76] and higher levels of stress and dissatisfaction regarding parenting [56, 76]. Furthermore, research has demonstrated an association between mental disorders such as BPD and hypermentalizing [77, 78], potentially resulting in an elevated sensitivity in the perception of negative emotions or malevolent attributions of these emotions in their children. The elevated utterances with negative valence in M-BPD, particularly in relation to themselves, are also consistent with findings from studies on autobiographical memory. These studies have indicated that individuals with BPD recall a greater number of negative events [79] and exhibit more anger [72]. It is noteworthy that the negative valence of maternal comments on their child’s activity, as assessed during an interacting task, did not differ between M-BPD and M-HC in a previous study [24]. This suggests that elevated negative representations may not necessarily transfer to the utterances directed towards the child. Consequently, future studies should investigate the impact of negative mental state references in M-BPD on parenting behavior. Finally, the reflection task may also be interpreted as indicative of a negative reaction to the child as a consequence of the intergenerational transmission of symptoms. Children of M-BPD have been observed to display observable abnormalities in their interactions and behaviors as early as the first months of life [80, 81], exhibit heightened negative affectivity and deficits in self-regulation [82], and display increased psychopathological abnormalities (Derhard R, Bunz M, Seehagen S, Schneider S: Mental health and temperament in young children of mothers with borderline personality disorder, in preparation) compared to children of mentally healthy mothers. Nevertheless, it should be noted that children of depressed mothers also demonstrate heightened negative affectivity and difficulties in emotion regulation [83], while mothers in the clinical control group in our study did not exhibit greater negativity in mental state references. It is recommended that future studies include these child-related variables and other factors that may influence negativity in mental state speech in their analyses.

#### Mind-mindedness and child-related mental state speech

In contrast with the findings of Schacht, Hammond [25], who reported less references to mental states among M-BPD compared to M-HC when talking about their child, the present study observed no difference between the two groups in their mind-related speech referring to their child. This discrepancy may be partly attributable to differences in child age across studies, as the younger average child age in our sample, particularly among M-HC, may influence mothers’ mind-mindedness differently [84]. In line with this assumption, we observed a small positive association between the use of child-references and child age. This finding may reflect the increasing verbal and cognitive abilities of older children, which provide parents with more explicit cues about their child’s mental states, thereby eliciting more mind-related descriptions from parents during the interview [22]. Since children of M-BPD were significantly older than those of M-HC, potential group differences may have remained undetected.

On the other hand, previous studies have demonstrated adequate test-retest reliability and reported no systematic age-related differences in representational mind-mindedness across a broad age range (e.g., [26, 85, 86]). Additionally, the wide age range of children in our study is comparable to that of prior research on representational mind-mindedness. Nevertheless, the potential influence of child age on the use of child-references should be considered a limitation of the present study, warranting further investigation with more age-homogeneous samples.

However, the absence of group differences in mental attributes utilized in reference to the child is consistent with research on the observational approach [24], reporting no difference in the quantity of mind-related speech directed towards 12-month-old infants between M-BPD and M-HC, but only in the quality with respect to an incorrect interpretation of the child’s behavior. It is crucial to acknowledge that the evaluation of mind-mindedness through the interview method does not encompass an assessment of qualitative aspects such as the appropriateness of the mind-minded comments in relation to both the child and the self. In contrast, the mind-mindedness indices are designed to record, in particular, the spontaneous tendency to reflect on mental content during the interview when the child is not present. Therefore, we can only gain insight into how mothers reflect on their children, potentially prompted by specific situations and interactions with their children that elicit reflection on the current situation and their child’s behavior.

Based on this limitation, it is also possible that existing differences in mind-mindedness between mothers with mental health issues such as BPD and healthy mothers might not be adequately depicted using an operationalization approach reliant on quantities, given the complex nature of these phenomena. Consequently, the thoughts, feelings, or needs of the child expressed by M-BPD may be misinterpreted, resulting in equally often but inappropriate comments about the child’s mental states. This phenomenon is consistent with the concept of increased hypermentalization [77]. Our findings on elevated pre-mentalizing processing modes among M-BPD in comparison to mothers from both other groups and the conceptually related finding on reduced interest and curiosity about the child’s mental states evident among M-BPD support this assumption. It is possible that this was not reflected in their amount of child-related mental speech due to methodological discrepancies between the two measures (self-report in a questionnaire versus spontaneous tendency measured in an interview about the child and the mutual relationship).

The absence of group differences between M-A/D and M-HC aligns with prior research findings [25–27], which indicate no correlation between depressive symptoms and the amount of mind-related speech. Only one study reported a negative association between anxiety symptoms and mind-related speech [26].

#### Not-knowing stance

We observed no difference between the groups in their tendency to express their mental state references in a not-knowing stance. This finding contradicts the conceptually overlapping results from the PRFQ, which suggest that M-BPD are less likely to report certainty regarding their perception of their child’s mental states than M-HC. However, this discrepancy may reflect differences in the underlying constructs measured by the two operationalizations, as indicated by the non-significant correlations between them.

It is possible that lower certainty among M-BPD does not necessarily manifest in the style of language used, and thus the MM_nk_ index may not fully capture the degree of recognition of mental state opacity that could be reflected in the PRFQ results.

Specifically, low PRFQ_CMS_ scores may indicate genuine confusion or difficulty in accurately perceiving the child’s mental states, while high scores may reflect being overly certain. Thus, average PRFQ_CMS_ scores are considered most adaptive [15, 16].

In contrast, the MM_nk_ index may capture a more deliberate not-knowing stance, characterized by an awareness of the opacity of mental states and the inherent limits to their complete comprehension. Such a reflective stance is expected to manifest in the style of speaking about mental states, rather than merely indicating uncertainty or confusion. Supporting this interpretation, we found a small positive association between higher education levels and the MM_nk_ index. This finding also suggests that higher MM_nk_ values – or a more pronounced not-knowing stance during the interview – could reflect greater social learning or a more sophisticated, deliberate reflection process. Following this interpretation, however, M-HC who reported higher education levels than M-BPD, did not significantly differ regarding MM_nk_ levels. This may indicate that the MM_nk_ index captures different forms of not-knowing, which may not be adequately distinguished by education level alone.

More research is needed to clarify the predictors of MM_nk_ and PRFQ_CMS_, as well as their levels of functionality and dysfunctionality. Additionally, exploring potential interactions between educational background, PRFQ_CMS_, and the MM_nk_ index could provide further insights into the complex nature of this construct.

#### Mind-mindedness frequency scores

The use of relative scores is a recommended and widely utilized method for controlling for verbosity [31, 59]. In other studies (e.g., [60]), total scores are employed to quantify the extent of mental state speech received by the child. Accordingly, we investigated group differences by examining total scores of mental attributes utilized by mothers during the interview in exploratory analyses. While relative scores account for variations in verbosity and thus provide insight into how mothers allocate their focus in the interview, relative to the actual quantity of speech produced, frequency scores emphasize the actual quantity of speech produced by the mother.

With the exception of mind-related speech directed at the child versus the mother herself, the results were comparable to those obtained using relative scores, thereby supporting the findings reported above and challenging the assumption that proportion values artificially alter the ranking and categorization of the parent as high or low mind-minded [60]. It is noteworthy that M-BPD made more self-references than M-HC when controlling for verbosity, whereas M-BPD made fewer child-references than M-HC when analyzing the total number of mental attributes about their children. This suggests that M-BPD place a greater emphasis on their own mental states in relation to the child and their shared experiences, which lends support to the notion of a heightened self-focus in M-BPD. However, this does not necessarily imply that they engage in more self-referential speech than other mothers in absolute terms. In absolute terms, M-BPD appear to engage in child-related mental state speech less extensively than M-HC, which may reflect a reduced comfort with reflecting on their child. This finding is consistent with the reduced levels of interest and curiosity about the child’s mental states observed in M-BPD compared to M-HC in this study. Conversely, despite the overall reduction in speech, M-BPD allocated a similar proportion of mental state references to their child as M-HC. However, further investigation is needed to ascertain the impact of these findings on real-world interactions with the child.

### Association between PRFQ and adapted mind-mindedness

Although both assessments demonstrate conceptual overlap and focus on representational mentalization, the PRFQ relies on self-ratings, whereas the mind-mindedness interview measure is observer-based. Furthermore, the PRFQ prioritizes child-related mentalization, whereas the representational mind-mindedness measure employed here additionally targets self-focused reflections. The results indicate that both operationalizations target distinct facets of parental mentalization. Only negativity in emotional valence was associated with all three aspects of parental reflective functioning in the anticipated directions.

In contrast with the findings of Smith-Nielsen, Stuart [36], our study revealed no correlation between the mothers’ inclination to employ mental state speech and their self-reported interest and curiosity regarding their child’s mental states. This discrepancy may be attributed to methodological differences, as our study employed an interview setting, whereas Smith-Nielsen, Stuart [36] analyzed mind-related speech during a free play session between mother and child. In general, studies assessing mind-mindedness in a free play task yielded inconclusive results regarding the association with parental reflective functioning (e.g., [8, 19, 35]). The absence of a link between mind-related speech in the interview and the three aspects of parental reflective functioning is also consistent with the frequently discussed “competence-performance gap”. This suggests that there is no inherent association between the ability to recognize mental states and behavior that is oriented towards the mind [11], providing further evidence for the idea of distinct concepts and measurement approaches. Furthermore, it can be assumed that additional factors such as variations in actual stress levels during the interview [59] are involved, contributing to variations in mind-mindedness that a questionnaire would not detect.

In our adaptation of the mind-mindedness coding manual [31], we introduced new dimensions, targeting additional aspects of mind-related speech that we consider potentially relevant for clinical samples. Our findings revealed a significant correlation between maternal negativity in their mental state speech about themselves and their child, and their self-reported extent of non-mentalization modes. This is consistent with the tenets of mentalization theory, which posits that non-mentalization modes are associated with malevolent attributions [16] that may give rise to negativity in mental state references. Furthermore, maternal negativity was linked to diminished certainty regarding their child’s mental states and a reduced interest and curiosity in these states. This may reflect aspects of hypomentalizing in mothers [16].

### Strengths and limitations

To the best of our knowledge, this is the first study on maternal mentalizing to compare M-BPD with a clinical and a healthy control group with a comparably large sample size, thus allowing statements to be made regarding disorder-specificity. Furthermore, we examined different facets of the multidimensional construct of parental mentalization through two distinct operationalization approaches, thereby providing further elucidation regarding the distinctions between them. Our adapted manual for coding mind-mindedness with the FMSS (Georg A, Zitzmann J: Manual Mind-Mindedness Kodierung von Reflexionsaufgaben mit dem FMSS, unpublished) enables a time-efficient assessment of mind-mindedness. Furthermore, it permits the examination of additional dimensions that may contribute to a more detailed understanding of the mentalization process, including the appraisal of one’s own mind and the mind of others [1], as well as emotional valence [40]. Previous evidence suggested that both are particularly relevant in clinical samples.

At the same time, several limitations of the study need to be considered. Firstly, further validation is required for the newly suggested indices and the assessment based on the FMSS in the context of the adapted manual. The utilization of mental state references may diverge from the observation of concrete behaviors when interacting with children. As an initial approach, we conducted a correlation analysis with the PRFQ. Nevertheless, further validation of the indices is required, including an examination of their associations with parenting behavior and the use of observer-based instruments. Secondly, as a consequence of the cross-sectional design of the study, the observed group differences may be attributable to additional variables. A key limitation is that sociodemographic variables were not controlled for in the group comparisons, despite significant group differences. Specifically, although the sample of M-BPD was representative of individuals with BPD [87], it lacked comparability to the M-HC group. Hence, the observed group differences in parental mentalization may be partially explained by sociodemographic factors. Additionally, M-BPD reported a greater number of indicators of socioeconomic burden, including unemployment and single parent status (Rosenbach C, Zitzmann J, Meyer C, Renneberg B: Parenting in mothers with borderline personality disorder - disorder-specificity and transdiagnostic aspects, in preparation). Prior research indicates that certain sociodemographic factors, such as socioeconomic background and parental education, may be associated with parental reflective functioning [15] and mind-mindedness [10, 59]. While a narrative review has provided mixed results concerning the relationship between mind-mindedness and educational attainment or other socioeconomic factors such as occupation [59], it remains important to consider these factors. In the present sample, significant correlations emerged between aspects of parental mentalization (particularly PRFQ subscales) and sociodemographic variables, such as child age and maternal education. However, due to violations of the assumptions required for conducting a MANCOVA, we were unable to statistically control for these factors. Therefore, we cannot rule out the possibility that the reported group differences in parental mentalization are at least partially influenced by sociodemographic factors. Future research should consider controlling for these sociodemographic variables to better differentiate the effects of psychopathology from those of sociodemographic influences. In addition, aspects related to parental mentalization, such as parenting stress, and subjective impairment due to psychopathology, should be examined. It would be beneficial for future studies to investigate longitudinally how specific deficits in parental mentalization develop dynamically during the first years of life, in both clinical and at-risk samples.

Thirdly, due to the aim of the main research project, we were only able to include female participants, which limits the generalizability of our conclusions. It would be beneficial for future studies to consider the perspectives of male caregivers as well.

Fourthly, it would have been valuable to analyze the clinical control group separately for depressive and anxiety disorders. However, this was not the primary aim of the original study. The high comorbidity within the clinical control group (*n* = 18) and the resulting small sample sizes for individuals with anxiety disorders (*n* = 21) and depressive disorders (*n* = 26) prevented separate analyses. At the same time, previous research has reported similar impairments in both disorders concerning parental reflective functioning, particularly in relation to pre-mentalizing [12, 19, 21], as well as mind-mindedness [12, 26]. Based on these results, we are confident that we did not overlook substantial differences between subgroups when merging the samples. However, the heterogenous clinical control group is still a limitation of our study, and future research would benefit from examining specific diagnostic subgroups separately.

Finally, it is essential to note that M-BPD must have undergone psychotherapeutic treatment at an earlier stage or are currently undergoing treatment in order to be included in the study. It seems reasonable to posit that existing mentalization deficits may have been improved following therapeutic interventions [7].

## Conclusion

Our study makes a significant contribution to the existing literature on maternal mentalization of mothers with BPD and anxious or depressed mothers compared to healthy mothers. In light of the findings which point to malevolent attributions and a negativity bias in parental mentalization, coupled with evidence of heightened self-focus in mental states among M-BPD, these aspects may prove beneficial in the diagnostic and therapeutic process, as well as in preventive interventions. It seems reasonable to posit that mothers with BPD are more likely to interpret their children’s mental states according to their own internal states and to talk more negatively. It may therefore be advantageous to encourage the taking of the child’s perspective and the promotion of interest and curiosity in the child’s mental states, as well as reflection on the processes of negatively biased attributions. Further research is warranted to clarify the influence of sociodemographic factors, such as maternal education and child age, and to determine how our findings translate to parenting.

## Data Availability

The adapted manual for coding mind-mindedness with the FMSS (Georg A, Zitzmann J: Manual Mind-Mindedness Kodierung von Reflexionsaufgaben mit dem FMSS, unpublished) is not publicly available but can be accessed upon reasonable request from the authors. The datasets used and/or analyzed during the current study are also available from the corresponding author on reasonable request.

## Abbreviations

BPD: Borderline Personality Disorder
PRFQ: Parental Reflective Functioning Questionnaire
PDI: Parent Development Interview
M-BPD: Mothers with Borderline Personality Disorder
M-A/D: Mothers with Anxiety Disorder or Depressive Disorder
M-HC: Healthy Mothers
FMSS: Five-Minute Speech Sample
PRFQ_PM_: Pre-Mentalizing Subscale of the Parental Reflective Functioning Questionnaire
PRFQ_CMS(−T)_: Certainty about Mental States Subscale of the Parental Reflective Functioning Questionnaire (transformed score)
PRFQ_IC(−T)_: Interest and curiosity Subscale of the Parental Reflective Functioning Questionnaire (transformed score)
MM_mental(_nr)_: Mind-Mindedness index (frequency score)
MM_self(_nr)_: Mind-Mindedness Self-related Index (frequency score)
MM_child(_nr)_: Mind-Mindedness Child-related Index (frequency score)
MM_nk(_nr)_: Mind-Mindedness Not-knowing Index (frequency score)
MM_neg(_nr)_: Mind-Mindedness Negativity Index (frequency score)
MM_pos(_nr)_: Mind-Mindedness Positivity Index (frequency score)
MM_neutral(_nr)_: Mind-Mindedness Neutrality Index (frequency score)
ICC: Intraclass Correlation Coefficients
MANOVA: Multivariate Analysis of Variance
MM_neg_child_: Mind-Mindedness Child-related Negativity Index
MM_neg_self_: Mind-Mindedness Self-related Negativity Index
